# Using a Responsive Feedback Approach to Develop and Pilot a Counseling Chatbot to Strengthen Child Nutrition in Rural India

**DOI:** 10.9745/GHSP-D-22-00148

**Published:** 2023-12-18

**Authors:** Namrata Tomar, Sriya Srikrishnan, Neal Lesh, Brian DeRenzi

**Affiliations:** a Dimagi India, New Delhi, India.; b Dimagi India, Mumbai, India.; c Dimagi USA, Boston, MA, USA.; d Dimagi South Africa, London, United Kingdom.

## Abstract

**Introduction:**

Nearly half of all deaths in children in India aged younger than 5 years are attributable to undernutrition. Reinforcing the caregiver's positive behaviors through multiple channels can lead to improved child nutrition outcomes. We describe the development and piloting of a chatbot to improve nutrition outcomes for children aged 0–12 months.

**Poshan Didi Chatbot Development Process:**

We engaged key stakeholders to understand existing interventions to improve nutrition outcomes and developed a theory of change that included an intervention that provides nutrition-related information directly to beneficiaries. A chatbot, Poshan Didi, was developed to provide individual counseling to mothers with children aged 0–12 months on age-appropriate, nutrition-related topics. The chatbot was piloted in Katni district from February 2019 to October 2019 in 2 phases to investigate the acceptability and feasibility of the chatbot (Phase 1, n=10 mothers) and to assess whether users would continue to be engaged and would engage with both the automated content as well as through free form questions (Phase 2, n=100 mothers). Quantitative and qualitative data were collected in focus group discussions with health care workers and mothers (n=4) and interviews with mothers (n=26).

**Results:**

Mothers viewed the chatbot as being a private channel to discuss topics and referred to Poshan Didi's persona as being knowledgeable. Eighty percent of users replied at least once to a chatbot-generated message. Sixty-four percent engaged beyond the standard chatbot content by discussing at least 1 issue with a nurse who responded to escalated messages.

**Conclusion:**

This work demonstrates how chatbots could create multiple interaction touchpoints between caregivers and health care workers to increase caregivers' access to age-appropriate nutrition counseling and information. The study shows the value of using the responsive feedback approach in the chatbot design and implementation to improve the efficacy of the digital tool.

## Abstract (precis)

The authors report on developing and testing a chatbot to facilitate additional client contact points for information and counseling to improve child nutrition outcomes in rural India.

## Teaser

**Key Findings:**

- Dimagi iteratively built and tested the Poshan Didi chatbot to facilitate direct-to-client engagement that yields improved parent awareness and knowledge of nutrition-related information.
- Mothers of children aged 0–12 months who tested the Poshan Didi counseling chatbot were receptive to the chatbot, appreciated the fast response time, and found the information they received useful.

**Key Implications:**

- Client-centered care benefits from additional contact points and follow-ups with the health system.
- Chatbots can be an appropriate health counseling tool for the future.
- The successful use of a sequential design process based on a responsive feedback approach has positive implications for future demonstration projects and their scale-up, specifically in the public health space.

## INTRODUCTION

According to the World Health Organization, 5.2 million children aged younger than 5 years die globally, mostly from preventable and treatable causes. Of these, almost 45% of deaths are caused by nutrition-related factors. India accounts for the second-highest number of deaths of children aged younger than 5 years.^1^ Routine household behaviors (e.g., diet and hygiene practices) greatly influence the nutritional status of children, which, in turn, emphasizes the need for continuous, age-appropriate counseling as a vehicle for driving behavioral change.^2^ Counseling that is delivered regularly and with the proper degree of quality can reinforce positive behavior, leading to improved child nutrition outcomes.^3^

Over the last 10 years, equipping community health workers with digital platforms has offered a major change in how global health can operate by providing scalable and efficient tools to improve service delivery. In 2018, India launched POSHAN Abhiyaan, the National Nutrition Mission, as a centrally sponsored scheme focused on alleviating the country's malnutrition challenges. The Integrated Child Development Services-Common Application Software (ICDS-CAS) was designed to equip community health workers, including Anganwadi workers (AWWs), with smartphones to strengthen service delivery and improve nutrition outcomes in the POSHAN Abhiyaan program through effective real-time monitoring and timely intervention. At the time of our work in 2018, ICDS-CAS was deployed in 550 districts, with plans to scale to the entire 1.4 million AWWs across all 36 states and territories, making it the largest ever mobile technology deployment in the world^4^ to augment the AWWs' data management capacity and efficiency.

In the next 10 years, an even greater change could occur by extending the health system's reach with “direct-to-client” innovations that enable users to access information and counseling on their smartphones at their convenience. There is an increasing trend in smartphone ownership and Internet connectivity. A study conducted by Deloitte in 2021 found that India had 1.2 billion mobile subscribers, of which 750 million used smartphones.^5^ The number of smartphone users in India is expected to reach 1 billion by the year 2026, most of which will be from rural areas at a compound annual growth rate of 6%.^5^ Of note, these positive trends in smartphone availability and ownership will need to be coupled with Internet penetration, which stood at 43% of the total population in 2020.^6^

Programs like ICDS-CAS rely on the AWW as the first touchpoint with the health system. The AWW-to-household ratio varies greatly, with the average being 1 AWW for a population of 400–800. There is an opportunity to augment AWWs' impact by engaging directly with beneficiaries to create additional touchpoints with the health system. We believe this will facilitate increased and continuous access to health-related knowledge and services through technology-based interventions, which have been primarily targeted toward health care workers.

A chatbot is a computer program that can talk to people through voice or text prompts similar to a human conversation and allows people to find information or access online services.^7^ Chatbots are rapidly gaining momentum due to breakthroughs in natural language processing, conversational artificial intelligence techniques, and the spread of low-cost Internet access. Wysa, a chatbot focused on improving mental health, reached more than 3 million users.^8^^,^^9^ Big Sis, which offers girls in South Africa a private and accurate channel to discuss topics they could not discuss elsewhere—such as sex, sexually transmitted infections, and HIV prevention—and points them toward services, had 85,000+ girls interested in its prototype on Facebook.^10^^,^^11^ Girl Effect has subsequently expanded its activities to India with the Bol Behen program, which was first launched in 2020.^12^ As programs and governments strive for universal health coverage, there is an increasing need to support more person-centered care. Chatbots can drive the next generation of digital technology, helping to reduce inequities in care.

Chatbots can drive the next generation of digital technology helping to reduce inequities in care.

Given the evidence for influencing positive household behavior through the reiteration of counseling messages, we aimed to test the feasibility and acceptability of increasing user engagement through a chatbot to influence behavior change that improves nutrition outcomes. The chatbot was developed using a responsive feedback (RF) approach that uses timely assessments, providing actionable feedback for implementers to integrate for improvements to help achieve the intended outcomes.^13^ Dimagi, a social enterprise technology company, used an iterative approach to build and test Poshan Didi (“nutrition sister” in Hindi), a semiautomated chatbot on a mobile messaging platform, to deliver timely individualized counseling information on age-appropriate, nutrition-related topics to mothers (the most common primary caregivers) with children aged 0–12 months. The chatbot design and development process incorporated 3 core RF characteristics that allowed it to be agile and adaptive during the pilot (Box).

BOX Core Responsive Feedback Characteristics of the Poshan Didi Development Process

- **Theory of change**. Clearly articulating a theory of change encourages stakeholders to agree on how the intervention will affect positive change and aims to surface assumptions to be tested during the deployment and strengthen the long-term theory of change.
- **Continuous testing**. Continuous testing of the implementation is necessary to detect deviations from expected outcomes, measure and respond to assumptions being tested, and avoid and identify implementation failures sooner.
- **Stakeholder engagement**. Active engagement with key decision-makers ensures that learnings are shared across all stakeholders and that the intervention is adapted in real-time with relevant inputs to correct theory and implementation failures that arise.^10^

**FIGURE 1:**
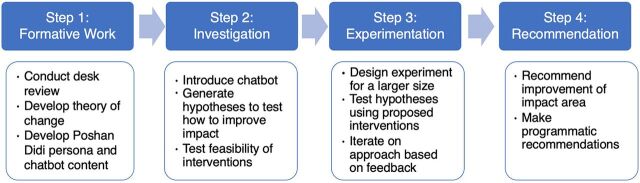
Poshan Didi Chatbot Development and Testing Process Steps

**FIGURE 2:**
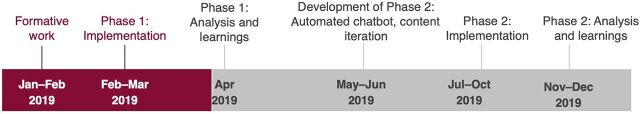
Poshan Didi Implementation Timeline

We provide an overview of the design and implementation process of Poshan Didi (Figure 1 and Figure 2) and elaborate on the feedback that was incorporated for its development.

## POSHAN DIDI CHATBOT DEVELOPMENT PROCESS

### Formative Work: The Next Generation of Digital Development

Initial site visits during the course of the ICDS-CAS program and a desk review of existing programs found that several interventions have been implemented to improve the AWW's knowledge and service delivery of health and nutrition counseling that could have impacted the adoption of positive health behaviors at the household level.^14^ However, an unmet need existed for additional contact points and follow-ups between health systems and their clients beyond what the understaffed health workforce could provide during in-person sessions. This was confirmed during conversations with AWWs to understand the reality of their work in this setting.

**FIGURE 3:**
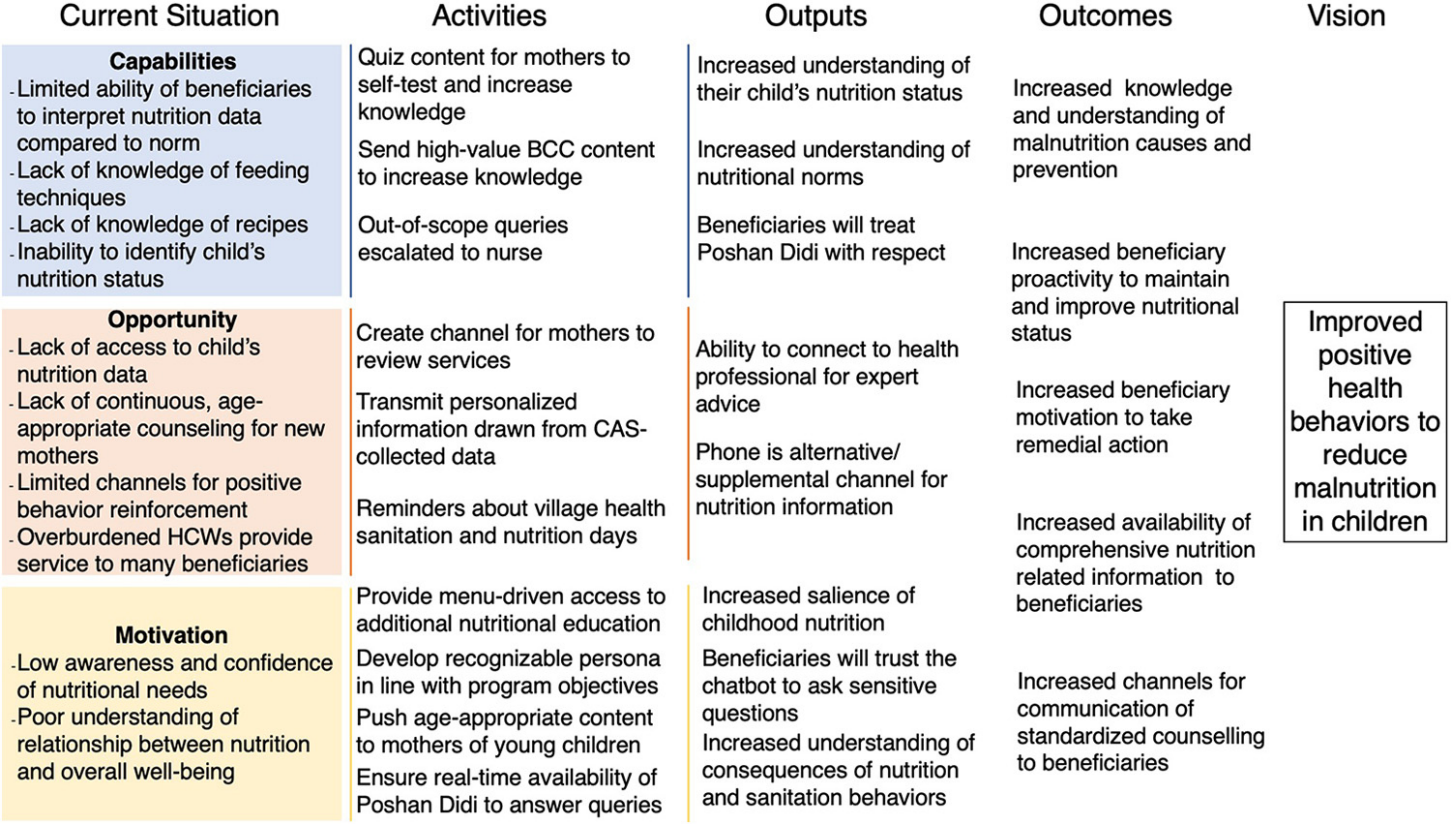
Theory of Change for Poshan Didi Chatbot to Improve Child Nutrition Abbreviations: BCC, behavior change communication; CAS, Common Application System; HCW, health care worker.

Based on the formative work done, a theory of change (Figure 3) was developed and subsequently updated to reflect the project team's experiences.

An initial version of Poshan Didi's persona was developed for feedback before Phase 1. Through a collaborative exercise during conversations with mothers—where they expressed their impressions of who Poshan Didi would be—we further refined the persona to better embody the cultural context of the community being addressed and the channel being used to introduce the tool (i.e., through existing AWWs). Some of the characteristics chosen for the Poshan Didi persona included being less authoritative than the AWWs, friendly and familiar (represented by similar clothing worn by women in the community), about the same age range as mothers being enrolled from the community, knowledgeable about positive health behaviors and practices, and a proponent of the counseling messages delivered by AWWs and government officials.

Messages were developed and delivered specifically for the mothers/primary caregivers with children within 2 age groups—0–6 months and 6–12 months. Content was adapted from publicly available incremental learning modules, which were developed by the government and were used during initial AWW training. This was done to reinforce existing health messaging and ensure alignment between the information that users received from different sources. The initial script was adapted in real time by the project staff based on the responses received from the users, allowing content to be continually refined during Phase 1 of the pilot testing and iterated further in Phase 2. Modules on kangaroo mother care and exclusive breastfeeding were available only to mothers in the 0–6 months group. Modules on complementary breastfeeding, complementary feeding, and complementary feeding recipes were available only to mothers in the 6–12 months group. All mothers received content from modules on sanitation, commonly occurring diseases in infants and young children, feeding techniques, lactating mother nutrition, and feedback on AWW services by the mothers.

**FIGURE 4:**
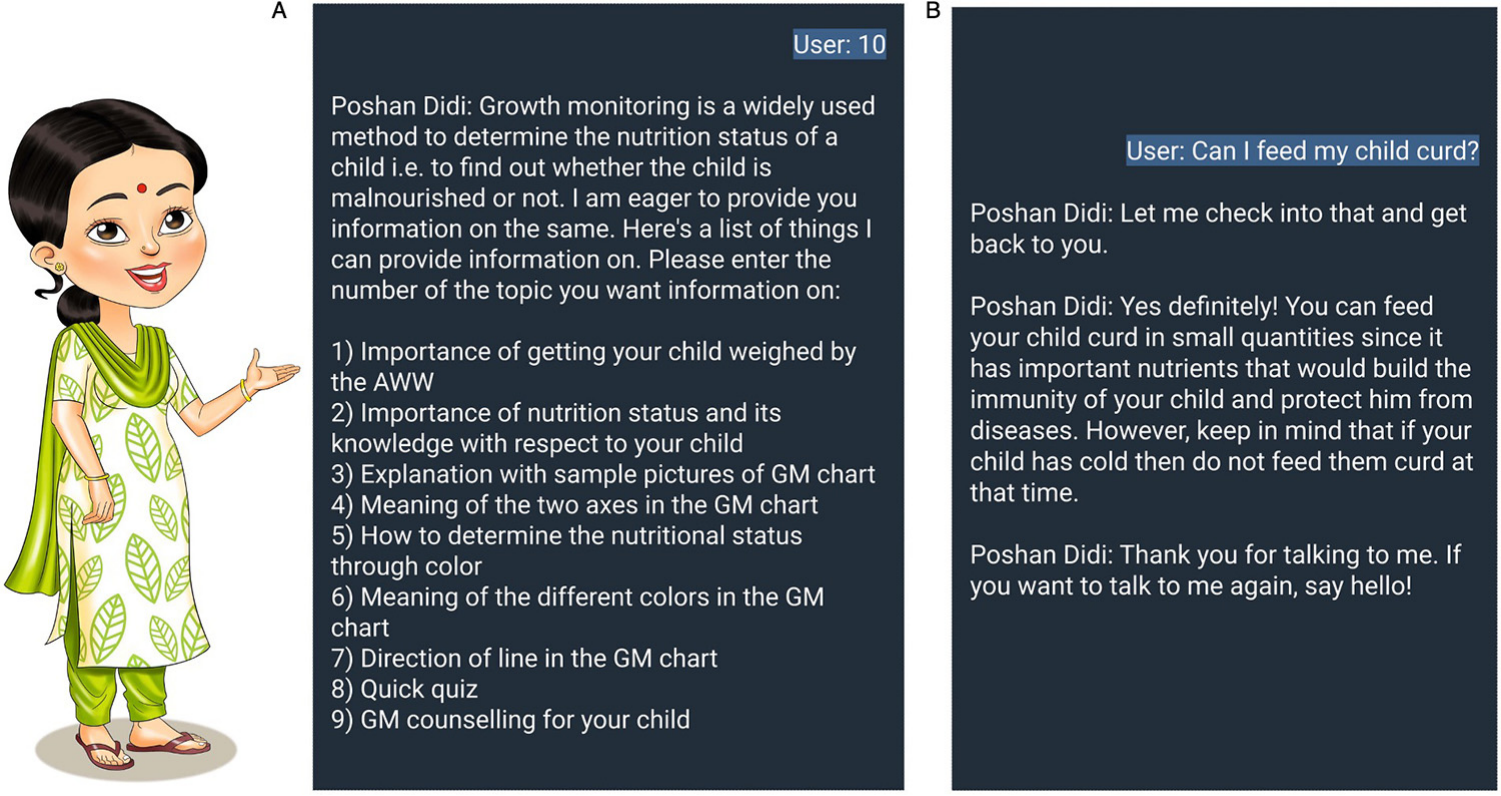
User Interface of Poshan Didi Chatbot

The Poshan Didi workflow and script were developed using Google Sheets and the TextIt platform, which is a hosted version of RapidPro that is maintained by Nyaruka. The “flow” was then exported, and custom prototype software was developed to support maintaining the state of each user during their interactions (i.e., where, within the hierarchy of menus and content, the user was at any given moment). The software would determine the next appropriate state—content or menu—based on an incoming message from a user and the current state of their interaction. After 30 minutes of inactivity, the state would reset, and Poshan Didi would send a message to the user thanking her and encouraging her to message “hello” to begin interaction again (Figure 4). A complementary web-based system was also developed to support messages escalated to the nurse.

The initial manual testing in Phase 1 of the pilot testing was done on WhatsApp, with 1 member of the project team directly messaging caregivers on WhatsApp, simulating the experience of interacting with an automated chatbot system. Later, in Phase 2, the automated prototype was developed and intentionally created to be platform agnostic. During the deployment, the Telegram platform was used extensively; however, Poshan Didi was also briefly tested on WhatsApp with 5 users. At the time of the work, WhatsApp instituted several limitations that made it difficult for governments and organizations to routinely engage with end users. As a result, those data are not analyzed in this article. The Poshan Didi code is open-sourced and unmaintained in these 2 repositories: https://github.com/dimagi/poshan-didi-client and https://github.com/dimagi/poshan-didi-server.

### Investigation: Assessing the Feasibility of Direct-to-Client Intervention

#### Pilot Site

Dimagi planned a 2-phased pilot in the Bahoriband block of Katni district (population: 1.29 million) in Madhya Pradesh, India (Table 1). The site was chosen for 2 reasons. First, good relations with the government officials in this area who showed a willingness to collaborate and test newer interventions were essential to the RF approach because it facilitated access to relevant and continuous feedback that could be incorporated into the implementation. Second, AWWs in this area showed high performance and had access to digital technology like ICDS-CAS. This site allowed the chatbot to be tested within an existing ecosystem of digital technology and an existing nutrition improvement program. The existing technology for data collection in the area and the collaborative nature of government officials who provided necessary permissions ensured the project team would have timely access to growth monitoring data to send to the mothers.

**TABLE 1.** Pilot Testing of Poshan Didi Chatbot, Madhya Pradesh, India

Pilot Phase	Users, No.	Sample Bias	Engagement
1	10	Users are manually chosen	Medium: WhatsApp. The intervention interaction is manual (users engage with Poshan Didi, but a researcher reads and responds manually to user questions). Evaluation is purely qualitative.
2	100	Users are not representative of the larger population	Medium: Telegram. Intervention is increasingly automated. Results are increasingly quantitative, though not necessarily widely generalized.

The chatbot was introduced to users as a supplement to existing health services around nutrition and health for a newborn child, such as kangaroo mother care, exclusive and complementary breastfeeding, and growth monitoring.

The chatbot was introduced to users as a supplement to existing health services around nutrition and health for a newborn child such as kangaroo mother care, exclusive and complementary breastfeeding, and growth monitoring.

The pilot test was expected to provide insights into how to build a more effective chatbot, including the type of messaging to which mothers would be most receptive (e.g., pushing out information or interactive question and answer), how users would react to directly receiving their child's growth monitoring data, and their usage patterns with respect to message frequency and timing.

#### Phase 1: Test Acceptability and Feasibility of Intervention

Phase 1 pilot was conducted for 3 weeks from February 18, 2019 to March 3, 2019 and aimed to assess the acceptability and feasibility of the chatbot. During this phase, we explored 3 core questions (Table 2): (1) How will users receive the chatbot? (2) How will users respond to incoming messages? and (3) How will the chatbot affect AWW services (quality and accountability)?

**TABLE 2.** Poshan Didi Chatbot Pilot Phase 1 Research Questions and Hypotheses

Questions	Hypothesis	Expectations From Learnings
How will users receive the chatbot?	The chatbot would be well received (i.e., users would find it useful to receive information through their phones via a persona-based, conversational interaction).	Understand smartphone penetration and usage Test feasibility of text-based messaging Explore initial reactions from beneficiaries on counseling information sent via technology Build content to include key topics of age-appropriate counseling
How will users respond to incoming messages?	Users would respond when they were engaged and interested.	
How will the chatbot affect AWW services?	Accountability of AWW services can be increased by asking users about recent AWW visits.	

Abbreviation: AWW, Anganwadi worker.

We selected 10 mothers to participate in Phase 1. Inclusion criteria were they had the ability to type on a phone keyboard, had basic literacy in Hindi, had a child aged younger than 1 year, were registered on ICDS-CAS, and owned or had access to a smartphone or a phone with WhatsApp. We grouped the mothers by their child's age: 0–6 months (n=5) and 6–12 months (n=5). The mothers provided their verbal consent to participate in the pilot, which was noted using a CommCare app for data collection.

Any adverse scenarios that required referral to health care services identified via the communication with the chatbot (such as a case where the child was diagnosed with pneumonia) were immediately flagged to local authorities for their attention.

This phase used WhatsApp to communicate with users manually, meaning that although users were informed that they would be engaging with the Poshan Didi chatbot, in fact, a project staff member read and responded to all messages manually, in adherence to the script development. Messages were sent with the user's name along with personalized charts built from data the AWW had collected through the ICDS-CAS. This process allowed for the refinement of content and aligned with the RF approach.

Phase 1 involved conducting baseline, midline, and endline interviews with all 10 participants. Each interview had at least 2 Dimagi staff present, with 1 interviewer and the other taking notes.

The interview responses, as well as the messages sent from users to the project staff serving as Poshan Didi, were used to answer our initial questions.

Aligned with the RF approach, incoming qualitative and quantitative data (surveys, user observations, and usage trends) were analyzed on a continuous basis to detect and correct any problems uncovered during the implementation with a focus on adhering to or adapting the theory of change. This included moving from a written to a menu-based system of interaction and adding further modules based on incoming questions, such as seasonal changes that affected a child's health.

### Experimentation: Poshan Didi Chatbot Implementation

Building on the Phase 1 results and the theory of change, we planned a second pilot Phase 2 to explore additional questions (Table 3): (1) We found mothers used very formal and polite language in Phase 1, demonstrating a high level of respect; how does users' respect compare during the semiautomated hybrid version of Poshan Didi? (2) How will users respond to incoming messages? Phase 2 was conducted for 3 months, from July to October 2019. The 2 tracks for the messages remained the same as the Phase 1 pilot (i.e., 0–6 months and 6–12 months). The content in Phase 1 was modified based on incoming questions from the participants during Phase 2, observations by the nurse, and feedback from district-level government officers who are responsible for decision-making for the nutrition program (Table 4). The chatbot was adapted to fit a menu-based interaction style, where users could choose options because their ability to type full messages was low.

**TABLE 3.** Phase 2 of the Poshan Didi Chatbot Pilot Phase 2 Research Questions and Hypotheses

Questions	Hypothesis	Expectations From Learnings
We found mothers used very formal and polite language in Phase 1, demonstrating a high level of respect; how does users' respect compare during the semiautomated hybrid version of Poshan Didi?	We would continue to see high levels of respect from users.	Evaluate response rates from beneficiaries based on content and message format
How will users respond to incoming messages?	At least 30% or more of the users will engage with the automated menu-based interaction menu system. At least 10% of users will use the nurse-escalation system to ask a free-form question. Messages that users receive but do not explicitly reply to could still be read by users and be valuable.	Understand the value of real-time responses to beneficiaries that can be achieved through automation Learn more about key counseling topics/queries that are relevant to mothers

**TABLE 4.** Changes Made During Phase 2 of Iterative Development of the Poshan Didi Chatbot

Responsive Feedback Analyzed	Implementation Change	Feedback Source
Multiple queries on nutrition for lactating mothers.	Added new module called “Nutrition for lactating mothers” to menu-based script.	Messages escalated to nurse
Seasonal changes during duration of deployment prompted a request for counseling information on commonly occurring childhood illnesses that cause setbacks in child nutrition.	Added new module called “Commonly occurring childhood diseases” to menu-based script.	Requested by local government partner
Users were not familiar with menu-based script and time gap between registration and push message meant users did not engage with system.	Built and introduced “echo mode” feature to increase familiarity with system and to practice typing numbers.^a^	Chatbot logs and midline interviews
Unintelligible queries (e.g., included random letters, incomplete words, and greetings) that did not necessitate a response.	Built “no reply” command that nurses could use to skip responding.	Messages escalated to nurse
Escalated queries that could be answered by menu-based script content that was available.	Built “state” command that nurses could use to redirect to menu-based script.	Messages escalated to nurse
User questions on capabilities of Poshan Didi and on existing content that was not yet shared.	Provided global menu with a complete list of modules to beneficiaries; project staff received additional training when necessary.	Midline interviews
Phone ownership and usage did not always correspond to user competency in using phone keyboard (∼70 women owned a personal phone).	Added keyboard training, including installation of local language for typing, to onboarding process.	Midline interviews
Users with children identified as severely malnourished did not access content on growth charts.	Sent push messages with growth charts to specific users.	Midline interviews

a Echo mode refers to a chatbot feature in which the bot validated the number entered by the user in the beginning for practice purposes. This had no impact on the actual conversation the participants had with the chatbot.

**FIGURE 5:**
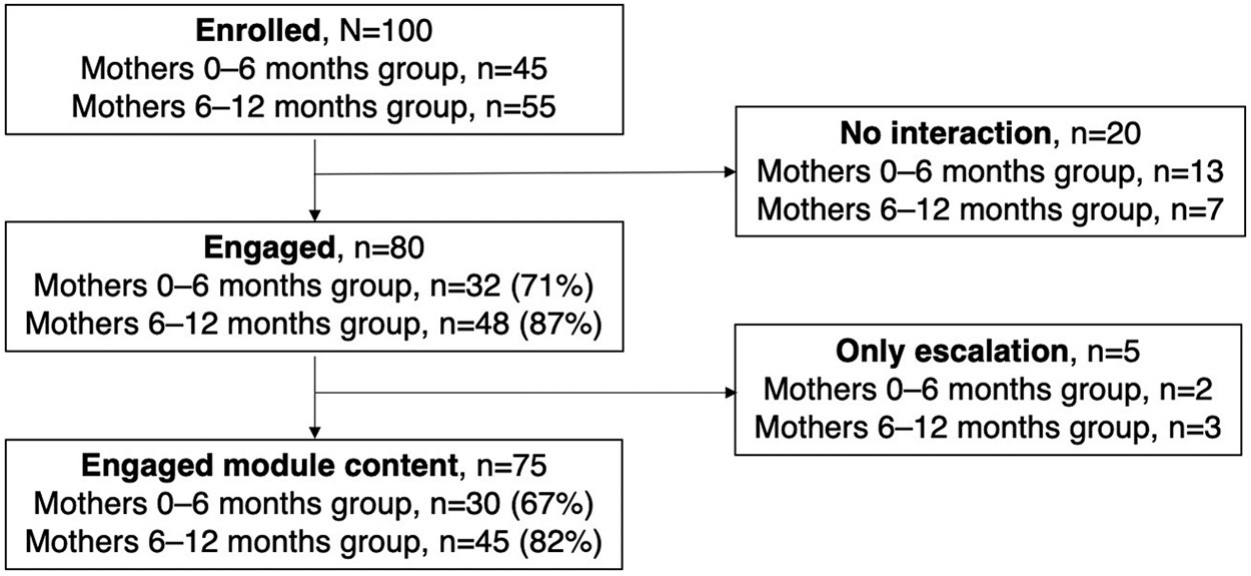
User Enrollment for Poshan Didi Pilot Phase 2 Implementation

We enrolled 100 mothers of children in 2 age groups: 0–6 months (n=45) and 6–12 months (n=55) (Figure 5). These caregivers were different from those who participated in Phase 1. The inclusion criteria were mothers who had the ability to type on the phone keyboard, had basic literacy in Hindi, had a child aged younger than 1 year, were registered on ICDS-CAS, and owned or had access to a smartphone. Similar to Phase 1, participants gave their verbal informed consent that was tracked using a CommCare application for data collection.

During this phase, a rudimentary semiautonomous chatbot could only understand numbers (typed as numerals or spelled out) corresponding to the topic number associated with the content in the topic-wise modules that were shared (Figure 4). Any message apart from numbers was considered an “out-of-vocabulary message” and was escalated to a live nurse to respond manually. This phase used the Telegram Messenger app.

Engaged users were those who interacted by sending at least 1 message to the chatbot. Although our original targets of 30% user engagement and 10% of users escalating to a live nurse were somewhat arbitrary, we chose these thresholds to reflect what we considered a standard rate of interaction based on another similar project (Table 3). However, after completing our work, we found some anecdotal evidence of industry norms for chatbots that support our initial targets. It has been reported that although the “better” chatbot experiences can achieve quite high response rates from users, “even the least favorable chatbot experiences are in the 35%–40% range.”^15^ We would expect public health messaging, which is informative and educational in nature, to perform worse than general commercial use of chatbots. With this in mind, the slightly conservative 30% user engagement reflects a lower bound. The 10% of users escalating to a nurse reflects our anticipation of fewer users requiring additional information outside of the content provided. We believed these thresholds would provide value to the user population.

#### Feedback Collection and Incorporation

The establishment of feedback loops and communication channels with key stakeholders, who included users, staff who observed the users, AWWs who interacted with the users, and district-level government officers, allowed for course correction and sequential development in a quick and agile manner. User feedback was collected through direct observation of messages sent, qualitative interviews, focus group discussions (FGDs), and continuous monitoring of usage data (Table 4).

All mothers in Phase 2 (n=100) completed a baseline survey over the course of the enrollment period in July 2019. This survey was done to understand users' demographic characteristics and analyze whether phone ownership affected usage and interaction. At midline (in mid-August to September), 26 in-person interviews were conducted. Interviews were conducted in Hindi, with 2 Dimagi project staff conducting the interviews. The interview selection criteria were based on the availability of the mothers at the time of the interview and the representation of different usage groups (high, medium, and low). At endline (October), we conducted a total of 4 FGDs: 2 FGDs with AWWs (5 and 6 AWWs, respectively) and 2 FGDs with users (6 and 5 mothers, respectively); interviews with mothers (n=26), and a survey of users. Only 76 mothers of the 100 enrolled in Phase 2 completed the endline survey; 1 participant was not present in the village, and 23 participants had limited/no interaction with the chatbot, so they declined to participate.

The questions from Phase 1 and Phase 2 were addressed through analysis of quantitative usage logs, as well as qualitative and quantitative data from baseline, midline, and endline surveys and FGDs. All FGDs had at least 2 Dimagi staff present, with 1 staff member serving as facilitator and the other taking notes. Qualitative data were derived from those FGD notes. We used methods such as card sorting^16^ and storytelling^17^ during the FGDs to engage users in a fun and interactive way and, most importantly, to reduce bias. Table 4 outlines the responsive feedback and changes made across both phases.

#### Ethical Approval

As this was a programmatic exercise, the project team did not seek formal ethical approval from an institutional review board before commencing the work. Approval to conduct the investigation was obtained from the relevant and appropriate programmatic leadership, specifically, the Director of the National Nutrition Mission for Madhya Pradesh State Administration and the Child Development Project Officer for Katni District Administration of the Department of Women and Child, Madhya Pradesh, India.

## RESULTS

### Perceptions, Engagement, and Value of Poshan Didi

#### Client Reception of Poshan Didi

Throughout both pilot phases, users viewed Poshan Didi as a confidential channel for information. In endline interviews, users expressed that they could discuss sensitive topics with Poshan Didi because they felt the conversations would remain private. In the FGDs, participants shared various interpretations of Poshan Didi's persona, including that Poshan Didi was an older, more serious woman because the participant believed that Poshan Didi was very knowledgeable. Another participant had the opposite response.

Users expressed that they could discuss sensitive topics with Poshan Didi because they felt the conversations would remain private.

*Poshan Didi is likely someone who is much younger because she [Poshan Didi] provided the latest information.* — Client, endline interview, Phase 2

*Thank you Didi for all this information. I only talk to you on such matters.* — Client, message sent to Poshan Didi, Phase 2

These responses were indications that Poshan Didi's persona played an important role, as it was both noticed and appreciated.

#### Client Response to Incoming Messages

Multiple mothers reported during in-person interviews that they had engaged with Poshan Didi—despite their interactions not appearing in the usage logs—suggesting the mothers may have engaged in mediated usage, in which another person used the Poshan Didi system on their behalf. Direct evidence of possible mediation was found: a user who had not used the system recounted a conversation—almost verbatim—that she had with the nurse, but in the usage logs, that conversation occurred with a different user. It seems plausible that a user acted as a mediator to the system, but we could not confirm this. It can be inferred that nonengaging mothers may still benefit from such an intervention in their community.

The automated, non-escalation path was a well-used pathway for information. Of the 100 enrolled, 80 mothers engaged (sent at least 1 message) with the chatbot and 75 mothers engaged with module content through the automated version of Poshan Didi (note that more than 1 message was required to navigate the menu system to get to module content), far exceeding our initial goal in the first hypothesis of 30% of users (Figure 5). This was also supported by qualitative interviews where users mentioned they found the content pushed out by Poshan Didi to be useful.

*I have gone through so much content, especially the complementary feeding recipes. Initially she [child] would not eat but I tried different ways and it worked.* —Client, endline interview, Phase 2

Nurse escalation—only available in Phase 2—proved to be a popular feature; 64% of users sent at least 1 message that escalated to the nurse (n=64). This far exceeded our target of 10% of users.

Most messages received a response in under an hour, with the vast majority in less than 5 hours. The study nurse did not work nights and weekends, leading to some longer delays. During the midline and endline interviews, many users reported that they appreciated the fast response time of Poshan Didi, including when a message was escalated to the nurse for manual reply. As previously noted, a user wrote that she appreciated receiving the family planning information she requested. The type of queries showed possible use cases for the chatbot beyond nutrition.

### Challenges

It is important to acknowledge that several women in the population from which the users were identified did not own a personal phone. If they had access to a shared household phone, they did not have it with them at the time of registration for the pilot. Consequently, registrations were completed using devices owned by the project team.

During testing, it was recognized that being able to use a phone and type did not necessarily translate to being able to interact with a chatbot. This led to keyboard training during registration and an “echo mode”—where Poshan Didi would respond to numbers that were sent to her—in the time between registration and the launch of Poshan Didi. The training aimed to ensure users knew how to easily send numbers from their keyboard and give them an opportunity to practice the skill with feedback. The training was well received, but very few users took advantage of the echo mode in between registration and the intervention to practice using it.

The mothers reported different barriers preventing their usage of the chatbot but most frequently cited a lack of time. Most mothers engaged with Poshan Didi in the afternoon and evening hours. They frequently reported having many competing household responsibilities that took up their time. Women who were registered on their household phones also did not have continuous access to phones. Several mothers mentioned that they preferred to use Poshan Didi only when they had a question or were facing some problems related to the health and nutrition of their child. As a consequence, they did not access the entirety of the content that was available in the chatbot. However, the mothers appreciated the always-on availability and immediate response available at a time of need.

*Time and availability is limited, home visit [by the AWW] does not happen every day and Poshan Didi can be present at all times.* — AWW, FGD, Phase 2

*No one knows how old Poshan Didi is and she talks in a very mature manner which is why the beneficiaries take her so seriously. We belong from their own community so they don't take us as seriously!* — AWW, FGD, Phase 2

Responses during FGDs with the health workers suggested that the tool would help reduce their workload and refocus their efforts on areas where more of their involvement was needed.

## DISCUSSION

### Recommendations for the Future of Direct-to-Client Innovations

Given the contexts where Poshan Didi was deployed and the promising response to the benefits of this type of tool, we believe that semiautomated chatbots supporting a health worker may be an effective avenue for health messaging. A considerable number of outputs that aligned with our theory of change (Figure 3) were observed during the short project period that directly influenced outcomes, such as an increase in mothers' knowledge regarding different nutrition-related topics (e.g., kangaroo mother care and complementary feeding) and proactively engaging with the chatbot to get more information. We are confident that interventions such as these will contribute to making positive behavioral changes to improve child malnutrition, as outlined in our theory of change.

Trends in Internet connectivity and smartphone ownership strongly support a shift toward more engaging, digitally supported interactions with clients. Although a lower level of smartphone ownership was seen among the women in the participant population at the beginning of the study, the iterative approach helped register newer phone users as participants during the pilot period. Additionally, the potential ripple effect that provided value to nonusers in the community is promising. Although this may have been a limitation to mothers who did not own phones, the chatbot was introduced as a supplemental channel of support in an area with AWWs. Additionally, the chatbot aligned with the same language and messaging used by AWWs to deliver nutrition-related information to families. This familiarity with the chatbot information that was shared and the chatbot's endorsement by AWWs, who have a close relationship with the community members, helped ensure the relevance and acceptance of the tool by users and enabled higher levels of usage.

In developing locally appropriate digital solutions, it is vital to facilitate collaborative decision-making, rapid prototyping, and user acceptance testing. Activities, such as card sorting and storytelling used during FGDs, engaged the users in an entertaining and engaging way and reduced bias. This approach is useful not only for designing applications but also for investigating user workflows and understanding their unique challenges. We engaged local stakeholders, such as first onboarding AWWs to the chatbot and using their network and in-depth understanding of the community to recruit mothers to the pilot in both phases, having regular check-ins with the block child development project officer to review script topics and take suggestions for new topics, and constantly sharing updates with stakeholders on the progress of the pilot phases.

### Value of the RF Approach in Direct-to-Client Tool Development

Several components of the RF approach that enabled continuous improvement of the tool and a better understanding of the factors that influence nutrition status can be used in developing future direct-to-client interventions.

#### Theory of Change

Ensuring that we were testing the tool in the context of the theory of change helped to focus the design of the tool on specific questions but still provided an avenue to understand areas for further experimentation. For example, a user's appreciative response to receiving family planning information she requested suggests that there are additional unmet information needs and that the tool may provide value in multiple contexts beyond malnutrition. However, including family planning content was out of scope for this project.

#### Continuous Testing and Monitoring

It is essential that data are constantly monitored during testing and that the implementation is measured to detect deviations from expectations. Data logs and midline interviews pointed to the fact that some women read the messages but never replied. This indicates that content should be designed to also be useful for users who may consume the information without engaging in conversation. Continuous monitoring is required to measure and respond to assumptions that are being tested and ensures that the organizations identify and avoid implementation failures as quickly as possible.

Continuous monitoring is required to measure and respond to assumptions that are being tested and ensures that the organizations identify and avoid implementation failures as quickly as possible.

#### Stakeholder Engagement

Productive engagement of stakeholders is of the utmost importance to keep the various actors—government, implementation partners, service delivery personnel, and users—informed to ensure effective feedback loops and course correction in a quick and agile manner. Due diligence and time should be kept for conducting market research on the tool. For example, if the direct-to-client intervention necessitates the requirement of a smartphone, then the ownership of the same should be assessed in the target population. The persona includes the name, icon, design, and text of the solution. These should be designed in such a way that the chatbot is viewed as a trustworthy source of information and a confidential channel of communication relevant to the context in which it will be used.

### Limitations

We acknowledge that we built a chatbot with a persona that mimics a human and that there are debates about the merits of this approach. While we recognize the value of this ongoing discussion, a more detailed discussion of the anthropomorphic design space and analysis of our decisions through that lens is outside the scope of this particular work and is left for future iterations. Another limitation of this study is that the goal of this work was to assess usability and acceptability through engagement and the new mother's experience with an escalating chatbot. Further work would need to establish the efficacy, cost-effectiveness, and equity of any intervention using a similar approach.

## CONCLUSION

The Poshan Didi chatbot that offers counseling and trusted nutrition information can help meet the need for additional client touchpoints and reinforce positive behavior changes that lead to improved child nutrition outcomes. An RF approach that iteratively incorporates feedback from users and other stakeholders throughout testing is useful for the development of such solutions.

## Notes

**Peer Reviewed**

**First published online:** November 16, 2023.
